# The Decline of Faith (Poetry)

**Published:** 1882-12

**Authors:** Paul Hamilton Hayne


### THE DECLINE OF FAITH.

(From the Century.)

As in some half-burned forest, one by one,
We catch far echoes on the dreary breeze,
Born of the downfall of its ruined trees—
While even through those that stand slow shudderings run,
As if Fate's hand were sternly laid thereon—
Thus, in a world smitten by foul disease—
That Pest called Doubt—we mark by sad degrees,
The fall of lordliest faiths that wooed the sun :
Some, with low sigh of parted bough and leaf,
Strain quivering downward to the abhorred ground;
Some totter feebly, groaning, toward their doom;
While some, broad-centuried growths of old Belief,
Sapped as by fire, defeatured, charred, discrowned
Fall with loud crash and long, reverberant boom !

Thus, fated hour by hour, more gaunt and bare,
Gloom the wan spaces, whence—a power to bless—
Unbourgeoned once, in grace or stateliness,
Some creed divine, offspring of light and air :
What then? Ah ! must we yield to bleak despair,
Beholding God himself wax less and less,
Paled in the skeptical flame-cloud's whirl and stress,
Till lost to love and reverence, hope and prayer?
O Man ! When trust is blind, and reason reels
Before some fiery, fierce Iconoclast,
Turn to thy Heart that reasons not, but feels ;
Creeds fall, shrines perish ! "Still" (her Instinct saith)—
"Still the soul lives ; the soul must conquer Death !
Hold fast to God, and God shall hold thee fast !"

PAUL HAMILTON HAYNE.

REMOVAL OF GREASE SPOTS.—Whenever oil of turpentine, benzole or ether is used to remove grease spots on cloth, the application should be made on the reverse side of the cloth, by moistening it with the solvent in a circle surrounding the spot, so as to approach it gradually, having blotting-paper in contact with the spot of grease, to absorb the fat immediately; otherwise the solvent will have the effect of spreading the grease over a larger surface instead of driving it out of the cloth. In the application of a hot iron to one side and blotting-paper to the other, the heat will drive the grease out of the cloth into the paper, because the fat has a tendency to move from the hotter parts toward the cooler.

